# Protective Effects of *Lactobacillus gasseri* against High-Cholesterol Diet-Induced Fatty Liver and Regulation of Host Gene Expression Profiles

**DOI:** 10.3390/ijms24032053

**Published:** 2023-01-20

**Authors:** Tianhua He, Nikita Lykov, Xu Luo, Huiling Wang, Zhanxiang Du, Ziyi Chen, Shitian Chen, Lin Zhu, Ye Zhao, Chimeng Tzeng

**Affiliations:** School of Pharmaceutical Sciences, Nanjing Tech University, Nanjing 211816, China

**Keywords:** fatty liver, *Lactobacillus gasseri*, high cholesterol diet, zebrafish, IL-17

## Abstract

Fatty liver is one of the most pervasive liver diseases worldwide. Probiotics play an important role in the progression of liver disease, but their effects on host regulation are poorly understood. This study investigated the protective effects of lactobacillus gasseri (*L. gasseri*) against high-cholesterol diet (HCD)-induced fatty liver injury using a zebrafish larvae model. Liver pathology, lipid accumulation, oxidative stress and hepatic inflammation were evaluated to demonstrate the changes in a spectrum of hepatic injury. Moreover, multiple indexes on host gene expression profiles were comprehensively characterized by RNA screening. The results showed that treatment with *L. gasseri* ameliorated HCD-induced morphological and histological alterations, lipid regulations, oxidative stress and macrophage aggregation in the liver of zebrafish larvae. Furthermore, the enrichment of the Kyoto Encyclopedia of Genes and Genomes (KEGG) pathway revealed that the core pathways of *L. gasseri* regulation were interleukin-17 (IL-17) signaling, phosphoinositide 3-kinase (PI3K)-AKT signaling pathway, the regulation of lipolysis and adipocytes and fatty acid elongation and estrogen signaling. The genes at key junction nodes, hsp90aa1.1, kyat3, hsd17b7, irs2a, myl9b, ptgs2b, cdk21 and papss2a were significantly regulated by *L. gasseri* administration. To conclude, the current research extends our understanding of the protective effects of *L. gasseri* against fatty liver and provides potential therapeutic options for fatty liver treatment.

## 1. Introduction

In recent years, fatty liver has become the most common chronic liver disease worldwide, with an occurrence rate of 25% [1]. The main feature of fatty liver is excessive liver fat accumulation (over 5%), which is associated with various liver diseases such as non-alcoholic steatohepatitis (NASH), liver fibrosis, cirrhosis and hepatocellular carcinoma (HCC) [2,3,4]. Fatty liver is also closely related to several adverse health conditions, including obesity, insulin resistance, type 2 diabetes and cardiovascular disease, etc. [5]. Nowadays, the options for the therapeutic treatment of fatty liver are still limited due to the multiple pathogenesis factors. The only effective strategies for the treatment of fatty liver are reducing the body mass index through exercising and dieting [6]. Thus, the search for new potential therapeutic agents is of paramount importance. The “two-hit” theory is the classical pathogenesis of fatty liver, which states that the hepatic overlord-lipid accumulation and oxidative stress are the two main factors contributing to the development of fatty liver [7,8]. Therefore, therapeutic strategies for fatty liver primarily focus on reducing hepatic lipid and oxidative stress.

Probiotics are considered to be live bacteria or micro-ecological regulators. When consumed in adequate amounts, probiotics can improve the health of the host by regulating the immune response and metabolism [9,10,11]. A large number of studies have confirmed the anti-obesity effect of probiotics, while other reports suggested that probiotics can reduce liver steatosis, which demonstrates that targeting microbiota for fatty liver seems reasonable [12,13,14]. Lactobacilli constitute an integral part of the normal gastrointestinal microbiota and are involved in improving host metabolism [15] and intestinal immune function [16,17,18], enhancing host resistance against infection and peritoneal macrophages [19]. The study of Wong et al. demonstrated that Lactobacilli treatment reduced liver steatosis and liver enzymes in the fatty liver animal model [20]. Therefore, the modulation of the gut microbiome with probiotics can be considered a promising strategy for the regulation of obesity-related comorbidities.

*Lactobacillus gasseri* (*L. gasseri*) is one of the predominant strains of lactic acid bacteria in the human small intestine, which has been reported to contribute to immune regulation, the prevention of bacterial and viral infection, reducing allergic symptoms, the inhibition of lipid absorption and has an anti-tumor effect [21,22,23]. *L. gasseri* can be isolated from the oral cavity, digestive tract, feces, vaginal cavity and areola and is considered to be a probiotic candidate with great research value [24,25]. A study analysis shows that *L. gasseri* SBT2055 can reduce postprandial lipid absorption and significantly reduce obesity [26,27]. However, the exact effects of *L. gasseri* and the mechanisms are still unclarified. In the present study, a fatty liver model based on zebrafish larvae was used to investigate the protective activities of *L. gasseri* against high-cholesterol diet (HCD)-induced fatty liver.

## 2. Results

### 2.1. Treatment with L. gasseri Ameliorated HCD-Induced Mortality and Morphological Changes of Zebrafish Larvae

**Figure 1:**
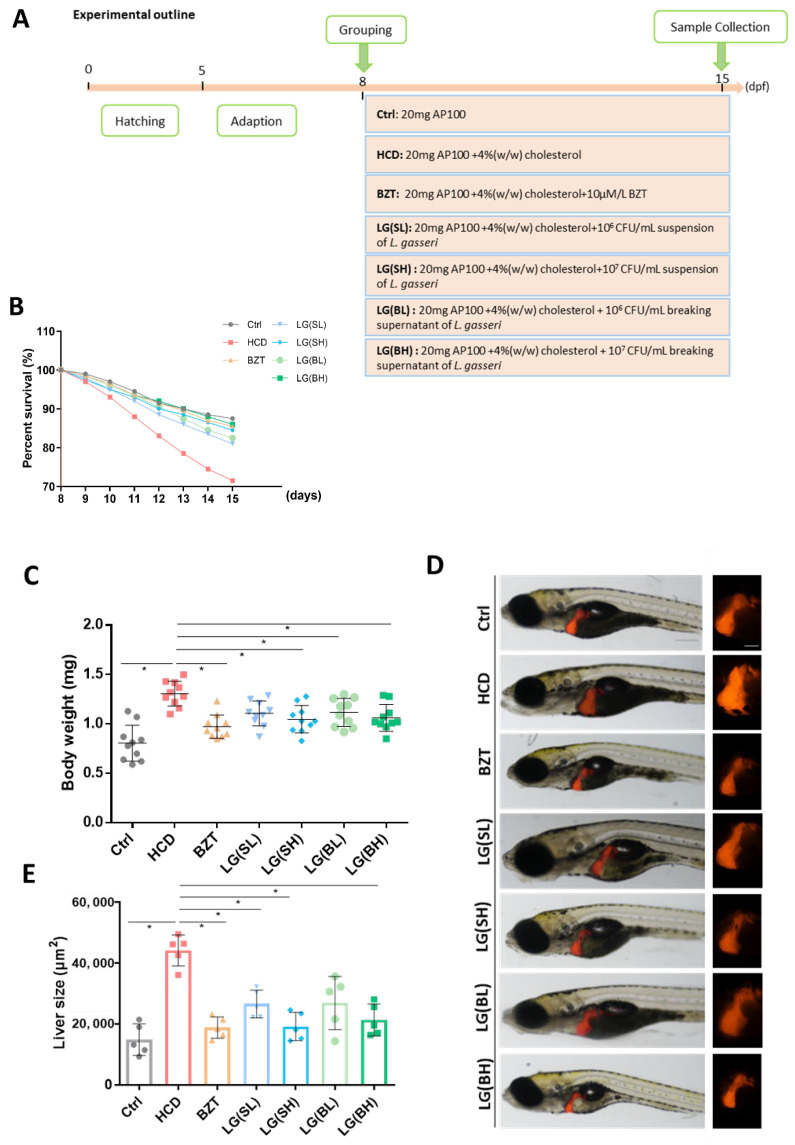
Effects of *L. gasseri* on high cholesterol diet (HCD)-induced mortality and liver morphological changes of zebrafish larvae. (**A**) Experimental outline of the feeding protocols. (**B**) Percent survival of zebrafish larvae from 8 to 15 days post fertilization (dpf), *n* = 30. (**C**) Body weight was measured at 15 dpf (*n* = 10). (**D**) Representative images of morphological alterations of transgenic zebrafish *Tg (lfabp10:dsRed)* liver under the same subdivided groups. The liver was labeled with DsRed. Scale bars = 100 µm. (**E**) Quantification of the liver size of *Tg (lfabp10: dsRed)* in each group, *n* = 5. Data were represented as mean ± SD. * *p* < 0.05.

The effects of *L. gasseri* on HCD (containing 4% cholesterol *w*/*w*) induced liver injury were tested on zebrafish larvae from 8 to 15 dpf with BZT as the positive control (Figure 1A). Four groups of *L. gasseri* administration were set with the cells (*L. gasseri* suspension) and the cell-free culture supernatants (*L. gasseri* breaking supernatant). After seven days of stimulation with HCD, the survival rate of zebrafish larvae was found to continuously decrease (Figure 1B). However, after one-week administration of *L. gasseri*, the decreasing survival rates were all relieved in both *L. gasseri* suspension (LG (SL) and LG (SH)) and *L. gasseri* breaking supernatant (LG (BL) and LG (BH)) groups. The percent survival curves of *L. gasseri* and BZT group were almost the same in variation trend (Figure 1B). Therefore, *L. gasseri* increased the survival rate of HCD-induced zebrafish larvae. The body weight of zebrafish larvae after the HCD treatment showed a statistical increase compared to the control. Notably, the groups fed with a high concentration of *L. gasseri* showed a significant reduction in body weight to a level comparable to that of the control and BZT groups, indicating that the *L. gasseri* had a weight-losing effect in HCD-induced larval zebrafish (Figure 1C). The transgenic fish *Tg (lfabp10: dsRed)* expressing red fluorescent protein (DsRed) in hepatocytes was applied with the same grouping to detect the gross liver morphology and liver size. As shown in Figure 1D, the area of red fluorescence was increased significantly in the HCD-treated groups as compared to the control. Notably, the size of red fluorescence in *L. gasseri* groups was reduced significantly compared to HCD groups. The quantitative statistics of the liver area presented a significant trend in a dose-dependent manner (Figure 1E).

### 2.2. L. gasseri Administration Attenuated HCD-Induced Fatty Liver on Lipid Accumulation and Histological Changes in Zebrafish Larvae

**Figure 2:**
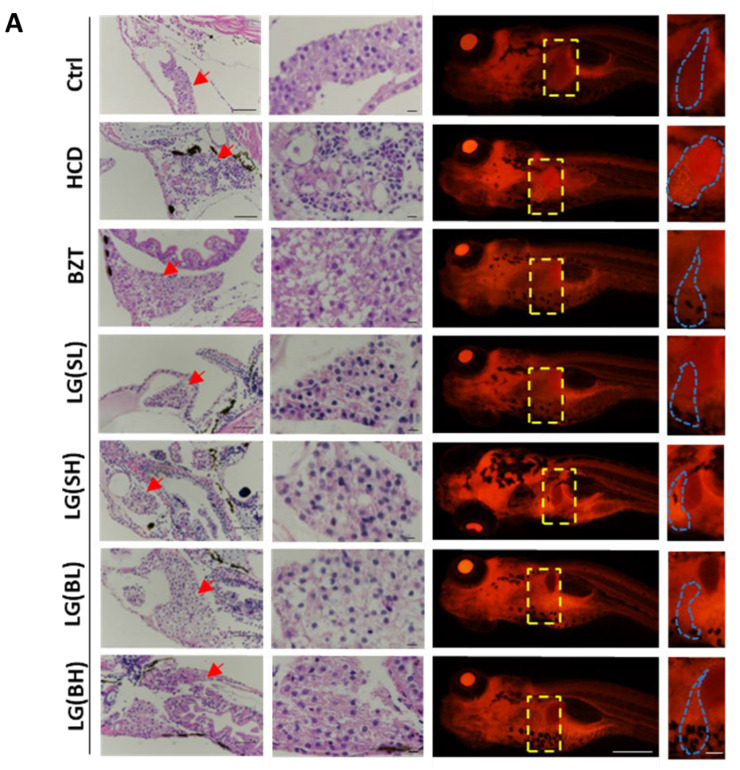

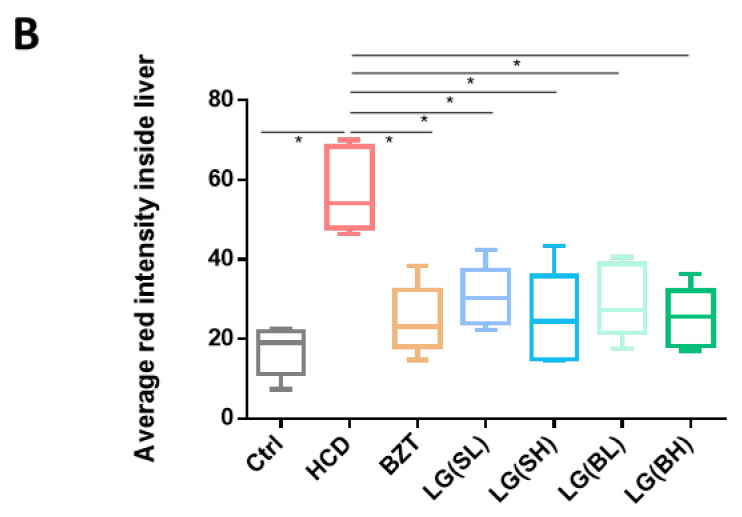
Effects of *L. gasseri* on HCD-induced fatty liver on lipid accumulation and histological changes in zebrafish larvae. (**A**) H&E staining and Nile red staining. Zebrafish livers were indicated with the red arrows in H&E sections and framed with a yellow box in Nile red sections. Scale bars = 100 µm. (**B**) Qualifications of Nile red staining by the relative red fluorescence intensity using ImageJ (*n* = 5). Data were represented as mean ± SD. * *p* < 0.05.

Subsequently, liver pathological changes in the liver of the corresponding groups were examined using H&E staining. In Figure 2A, zebrafish livers are indicated with red arrows. It could be observed that in normal liver tissue, the hepatocytes with clear polygonal shapes were joined together by a tight junction and with well-preserved cytoplasm and prominent nucleus. However, in the HCD-treatment groups, macrovesicular steatosis was obviously visible. The liver presented various tiny and large vacuoles, with loose cell-to-cell contact, dissociated and irregularly shaped cells and macrovacuoles. After *L. gasseri* treatment for one week, the deterioration of liver histological changes was obviously reversed. Moreover, the Nile red staining results showed that the average red fluorescence intensity in the liver was remarkably strengthened in the HCD treatment group as compared to the control (Figure 2B). However, the average intensity of the *L. gasseri* administered groups was comparatively weaker than the HCD groups. The improvement effects of various concentrations and different processed ways of *L. gasseri* on lipid deposition were almost equal to BZT, which revealed that *L. gasseri* might have a potential role in the alleviation of HCD-induced liver steatosis in neutral lipid deposition. 

### 2.3. L. gasseri Supplementation Alleviated HCD-Induced Fatty Liver on Oxidative Stress and Macrophage Aggregation in the Liver of Zebrafish Larvae

**Figure 3:**
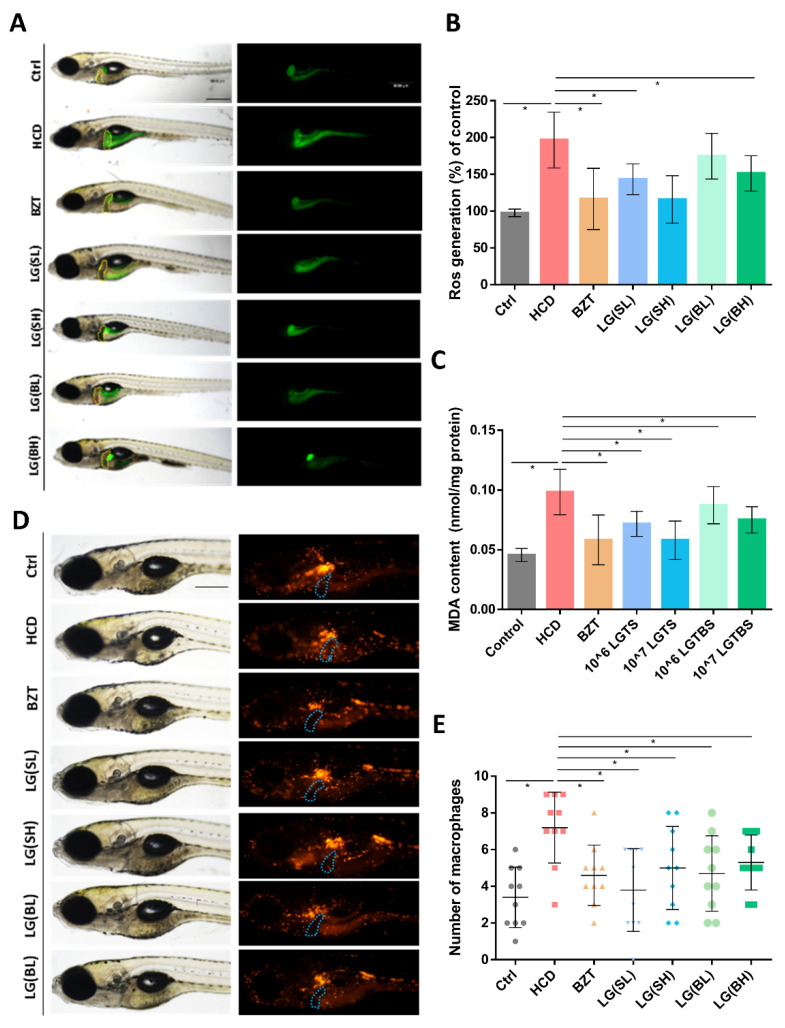
Effects of *L. gasseri* on HCD-induced oxidative stress and macrophage aggregation in the liver of zebrafish larval. (**A**) Reactive oxygen species (ROS) staining with 2′,7′-dichlorodihydrofluorescein (DCFH-DA). Liver was outlined with yellow dot circles. (**B**) Quantification of ROS staining by measuring the green fluorescence density of zebrafish liver (*n* = 10). (**C**) Detection of the contents of malondialdehyde (MDA) in vivo (*n* = 10). (**D**) Infiltration of macrophages in the liver was examined using the reporter transgenic line *Tg (mpx:mCherry)*. Macrophages were labeled with red fluorescence. Liver was indicated with blue dot circles. (**E**) Quantification of the number of macrophages in the liver of zebrafish (*n* = 10). Scale bars = 100 µm. Values were represented as mean ± SD. * *p* < 0.05.

To demonstrate the effects of *L. gasseri* on HCD-induced oxidant attack in zebrafish larvae liver, DCFH-DA, a fluorescent dye for ROS, was used to stain the oxidative stress product ROS in vivo. As shown in Figure 3A, the fluorescence of the liver (outlined with a dashed yellow line) was increasingly distributed in the HCD modeling groups as compared to the controls and was weakened after the treatments of *L. gasseri* in different ways. The quantification of green fluorescence intensity indicated that *L. gasseri* reduced the HCD-induced widespread ROS significantly (*p* < 0.05) (Figure 3A,B). Moreover, the activities of nonenzymatic antioxidant MDA were evaluated by biochemical assays. It was shown that the content quantification of the MDA presented a significantly higher level in the HCD treatment groups, while *L. gasseri* performed a remarkable function in reducing the levels of MDA in vivo (Figure 3C).

To further understand if *L. gasseri* had an effect on the inflammatory response caused by HCD, the infiltration of macrophages in the liver was examined using the reporter transgenic line *Tg (mpx:mCherry)*. The visualized results showed that macrophage infiltration was remarkably increased in the HCD treatment groups (Figure 3D). BZT alleviated HCD-induced macrophage aggregation in the liver of zebrafish larvae. Similarly, different forms and concentrations of *L. gasseri* treatments all decreased macrophage accumulation in the liver. The degree of macrophage infiltration reduction is analyzed in Figure 3E. All exhibited an increase in the number of liver macrophages after feeding with HCD, while the *L. gasseri* supplementation can restrain macrophage aggregation.

### 2.4. Transcriptome Profiling of HCD Revealed Hepatic Steatosis Injuries in Zebrafish Liver

**Figure 4:**
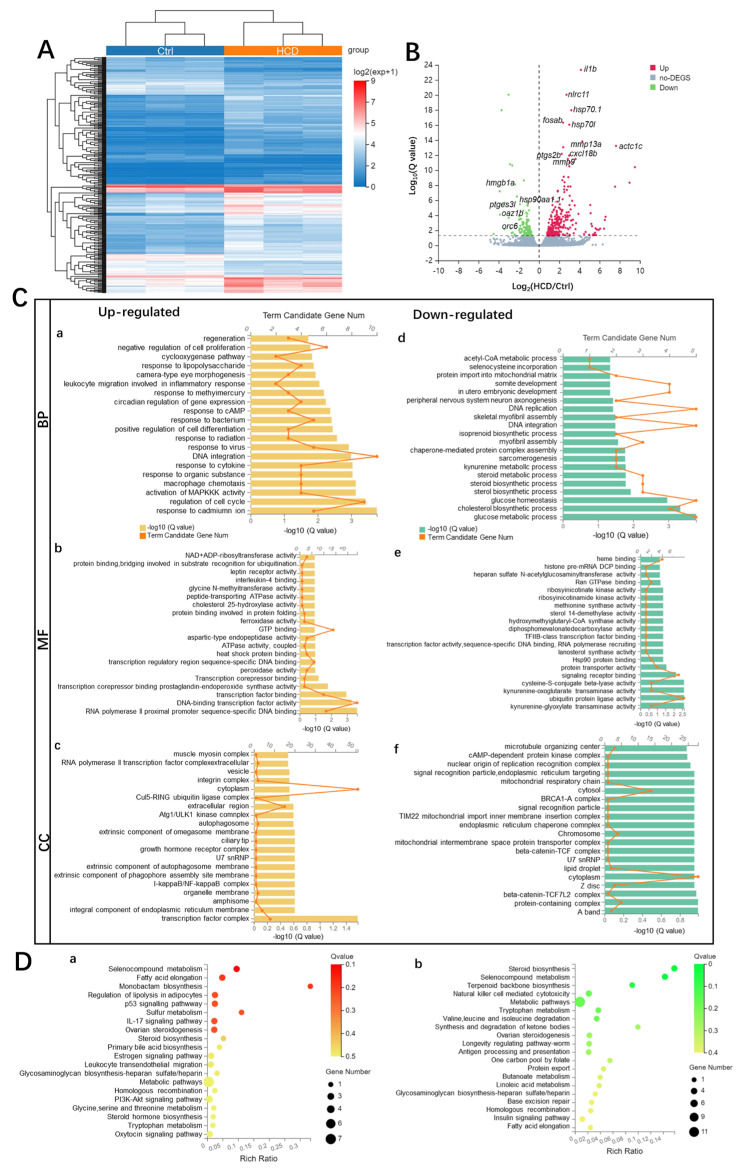
Transcriptome profiling of HCD-induced alternations of gene expression during liver injury in zebrafish larvae. (**A**) Hierarchical clustering of RNA-Sequencing data showed differentially expressed genes (DEGs) between Ctrl and HCD-induced groups (|log2FC| > 0, Q value < 0.05) by Pearson correlation method without mean centering. High and low levels of gene expression were represented by red and blue. (**B**) Volcano plots of identified DEGs of log2 (fold change) versus-log10 (Q value). The up-regulated DEGs are indicated with red dots, and the down-regulated DEGs are indicated with blue dots. Grey dots represent non-DEGs. (**C**) GO annotation, including biological process (BP) (**a**,**d**), molecular function (MF) (**b**,**e**) and cellular component (CC) (**c**,**f**) were characterized by DAVID analysis. (**D**) KEGG pathway analysis of DEGs. The top 20 enriched KEGG pathways associated with up- and down-regulated mRNA transcripts were presented in bar charts (**a**,**b**). Q < 0.05 were considered to be enriched.

To identify the potential molecular function of *L. gasseri* in cholesterol-induced high-fatty liver, the RNAs isolated from zebrafish liver of HCD, *L. gasseri* treatments and Ctrl with three biological replicates were sequenced, and transcript expression was comparatively analyzed mapping to the zebrafish RefSeq database. Compared with the vehicle control groups, a total of 400 differentially expressed genes (DEGs) were identified, including 276 up- and 124 down-regulated DEGs (|log2FC| > 0, Q value < 0.05). By hierarchical clustering based on DEG expression patterns, as shown in Figure 4A, the HCD were clustered in a similar relationship and well separated from all of the control samples (Ctrl), suggesting that there were substantial changes were caused by cholesterol induction in zebrafish liver. Volcano maps of the expression distribution of these DEGs are displayed according to their fold change, of which the top 15 functional DEGs were labeled (Figure 4B). 

To broaden the understanding of the functional categories of the DEGs, GO functional enrichment was characterized through DAVID analysis for annotation. The Q value was calculated using a hypergeometric test and the enriched term or pathway with Q < 0.05 was considered significant. The enrichment of the upregulated GO term showed that variations in DEGs linked with the biological process (BP) were significantly enriched in macrophage chemotaxis, leukocyte-migration-involved inflammatory response, the activation of MAPKKK activity, cyclooxygenase pathway and DNA integration (Figure 4(Ca)). In the matter of molecular function (MF), the DEGs of the enzymes responsible for lipid metabolism, such as cholesterol 25 hydroxylase activity, cytotoxicity oxidase enzymes, such as peroxidase, and ferroxidase activity were significantly enriched after HCD induction (Figure 4(Cb)). The cellular component (CC) was mainly enriched in the transcription factor complex (Figure 4(Cc)). Correspondingly, the down-regulated GO term showed that variations in DEGs associated with BP were mainly enriched in the glucose metabolic process, glucose homeostasis, cholesterol biosynthetic process and steroid and sterol biosynthetic processes (Figure 4(Cd)). MF were significantly enriched in enzymes, such as ubiquitin-protein ligase activity and sterol 14-demethylase activity (Figure 4(Ce)). With regard to CC, downregulated DEGs were significantly enriched in lipid droplet and mitochondrial intermembrane space protein transporter complex, etc. (Figure 4(Cf)).

DEGs were subjected to KEGG pathway analysis to identify the associated pathways and molecular interactions to further predict the molecular mechanism of cholesterol in the liver. Items with corrected Q < 0.05 were considered to be significant or enriched. The KEGG gene set biological process database (c2. KEGG. v4.0) from the Molecular Signatures Database was used for enrichment analysis. As shown in the green dashed circle of Figure 4D,E, the top 20 enriched KEGG pathways associated with up-and down-regulated mRNA transcripts were presented, respectively. The interleukin-17 (IL-17) signaling pathway, phosphoinositide 3-kinase (PI3K)-AKT signaling pathway and leukocyte transendothelial migration were highly related to HCD-induced liver inflammation reaction. In addition, the p53 signaling pathway, glycosaminoglycan biosynthesis–heparan sulfate/heparin and primary bile acid biosynthesis were also involved in liver hepatic steatosis processes. Moreover, HCD also affected fatty acid elongation, as well as steroid biosynthesis and ovarian steroidogenesis and selenocompound metabolism, implying that the HCD-induced hepatic steatosis injury might be associated with hormone regulation.

### 2.5. Potential Functional Mechanisms of L. gasseri on Gene Expression Profiles of HCD-Induced Hepatic Steatosis Injury

**Figure 5:**
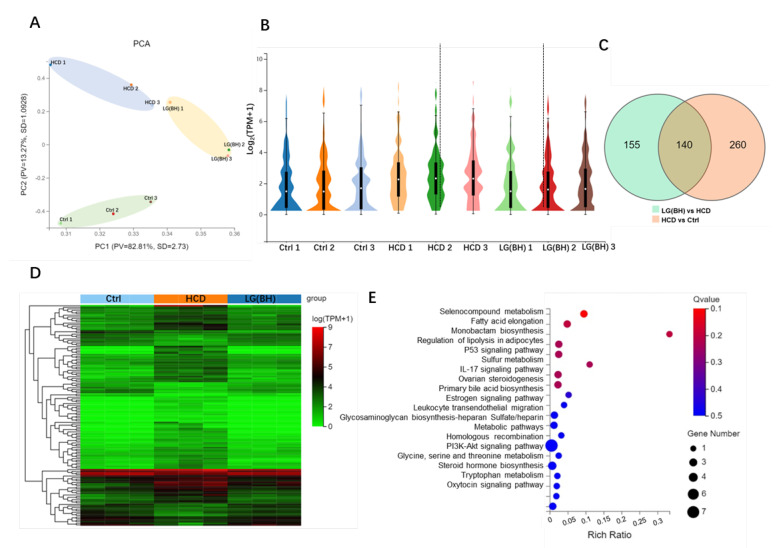

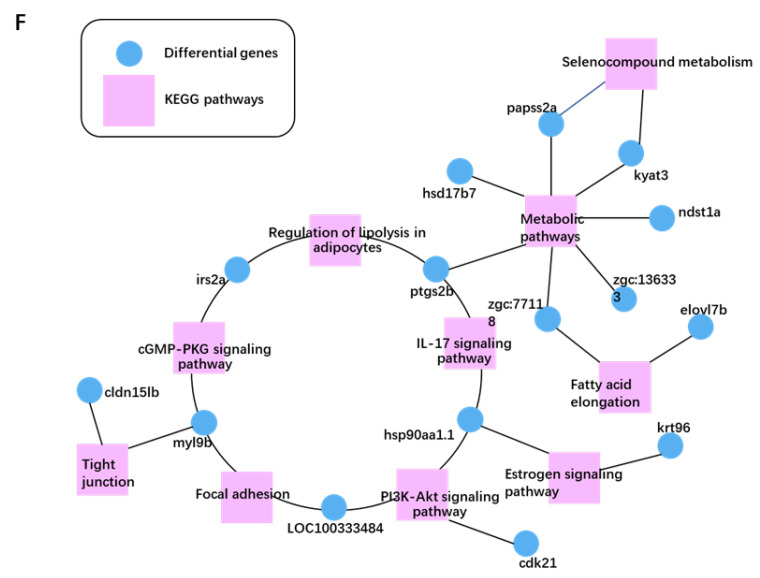
Transcriptomic analysis of gene expression pattern after *L. gasseri* administration. (**A**) Principal component analysis (PCA) of the samples, including Ctrl, HCD and LG (BH). (**B**) Violin plot displayed the distribution of log2 (TPM + 1) of gene expressed in each group. TPM, transcript per million. The white dot marks the mean value of the distribution, and the black bar corresponds to the 25th (bottom) and 75th (top) percentile range. (**C**) Venn diagram showed the overlapping DEGs between HCD vs. Ctrl and LG (BH) vs. HCD (|log2FC| > 0, Q value < 0.05). (**D**) Differential cluster heat map of fold change (log2 fold change) of the overlapping 140 DEGs using Pearson correlation method without mean centering. (**E**) KEGG enrichment of the overlapping 140 DEGs. (**F**) The top 10 KEGG pathway relationship network with the largest node connections number of genes. The correlated pathways and genes were connected by black edges.

To unravel the transcriptional programs regulated by *L. gasseri*, we further performed RNA-seq comparison analysis of Ctrl, HCD and LG (BH). In the principal component analysis (PCA), the samples of the same treatment group were clustered together, demonstrating a significant divergence (*p*  <  0.05) among the control, HCD and LG (BH) (Figure 5A). The violin plot displayed the distribution of log2 (TPM + 1) of gene expression, indicating the decreased gene expression of Ctrl and LG (BH) relative to HCD (Figure 5B). The Venn diagram in Figure 5C displayed that there were 140 overlapping DEGs between HCD vs. Ctrl and LG (BH) vs. HCD (|log2FC| > 0, Q value < 0.05). The differential cluster heat map showed the fold change (log2 fold change) of the overlapping 140 DEGs, the majority of which (106) were upregulated by HCD and downregulated after the treatment with *L. gasseri* (Figure 5D). This group included keratin 96 (krt96), histone deacetylase 10 (hdac10), elongases of very long-chain fatty acids 7b (elovl7b), insulin receptor substrate 2a (irs2a), glycine N-methyltransferase (gnmt), EH-domain-containing 1a (ehd1a), myosin, light chain 9b (myl9b), C-X9-C motif containing 1 (cmc1), Rab acceptor 1 (rabac1), claudin 15-like b (cldn15lb), membrane-spanning 4-domains, subfamily A, member 17A.9 (ms4a17a.9), pleckstrin homology-like domain family A member 2 (phlda2), Rho guanine nucleotide exchange factor (GEF) 3 (arhgef3l), prostaglandin–endoperoxide synthase 2b (ptgs2b), troponin I4b, tandem duplicate 1 (tnni4b.1), transmembrane protein 104 (tmem104), mesoderm-induction early response 1, family member 3 b (mier3b), cyclin-dependent kinase 21 (cdk21), potassium voltage-gated channel, subfamily H (eag-related), member 6b (kcnh6b), NADPH oxidase organizer 1a (noxo1a), actinin, alpha 2b (actn2b), phorbol-12-myristate-13-acetate-induced protein 1 (pmaip1), 3’-phosphoadenosine 5’-phosphosulfate synthase 2a (papss2a), ciliogenesis and planar polarity effector complex subunit 1 (cplane1), nectin cell adhesion molecule 4b (nectin4b), chemokine (C-X-C motif) ligand 18b (cxcl18b), integrin subunit beta 7 (itgb7), membrane-spanning 4-domains, subfamily A, member 17a.2 (ms4a17a.2), protein tyrosine phosphatase non-receptor type 21 (ptpn21). With regards, 34 DEGs were downregulated in HCD but upregulated after *L. gasseri* treatment, including ms4a17a.8, importin 13 (ipo13), N-deacetylase/N-sulfotransferase (heparan glucosaminyl) 1a (ndst1a), transcription factor 4 (tcf4), ubiquitin interaction motif containing 1 (uimc1), heat shock protein 90, alpha (cytosolic), class A member 1, tandem duplicate 1 (hsp90aa1.1), kynurenine aminotransferase 3 (kyat3), RNA-binding protein, mRNA-processing factor 2a (rbpms2a), reticulon 4a (rtn4a), solute carrier family 7 member 2 (slc7a2), ELMO-domain-containing 2 (elmod2), SET and MYND-domain-containing 2b (smyd2b), eukaryotic elongation factor, selenocysteine-tRNA-specific (eefsec), claudin 15b (cldn15b), hydroxysteroid (17-beta) dehydrogenase 7 (hsd17b7) (Supplement Tables S1 and S2).

The KEGGs of the overlapping DEGs were evaluated to gain detailed functional insights into the mechanism of the ameliorating effect of L. gasseri on HCD-induced fatty liver. It was shown that the fatty acids elongation, monobactam biosynthesis, the regulation of lipolysis in adipocytes were significantly associated with *L. gasseri* modulation. Leukocyte transendothelial migration was affected by *L. gasseri*, which was consistent with the results of macrophage migration (Figure 5E). The IL-17 signaling pathway, PI3K-AKT signaling pathway and oxytocin signaling pathway were also involved in *L. gasseri* regulation. Moreover, *L. gasseri* was active in the signaling related to steroids hormone biosynthesis, steroids biosynthesis, as well as an estrogen signaling pathway, ovarian steroidogenesis. It was shown that selenocompound metabolism was affected by *L. gasseri*, with the regulation of two key genes, papss2. Changes in primary bile acid biosynthesis and heparin, related to hepatic steatosis, were also moderated by *L. gasseri*.

The KEGG pathways of the 140 overlapping DEGs between HCD vs. Ctrl and LG (BH) vs. HCD were ranked by the number of genes on the pathway, and the top 10 pathways with the largest node connections number of genes were selected to construct the KEGG pathway relationship network (Figure 5F). Each node, indicated by pink squares, represented one KEGG pathway. The correlated pathways and genes were connected by black edges. The network was constructed based on the results of the KEGG pathway analysis and KEGG database, which indicated that the core pathways of *L. gasseri* regulation were IL-17 signaling, PI3K-Akt signaling, cGMP-PKG signaling, focal adhesion and tight junction, the regulation of lipolysis and adipocytes and fatty acid elongation, estrogen signaling, selenocompound metabolism and other metabolism signaling.

**Figure 6:**
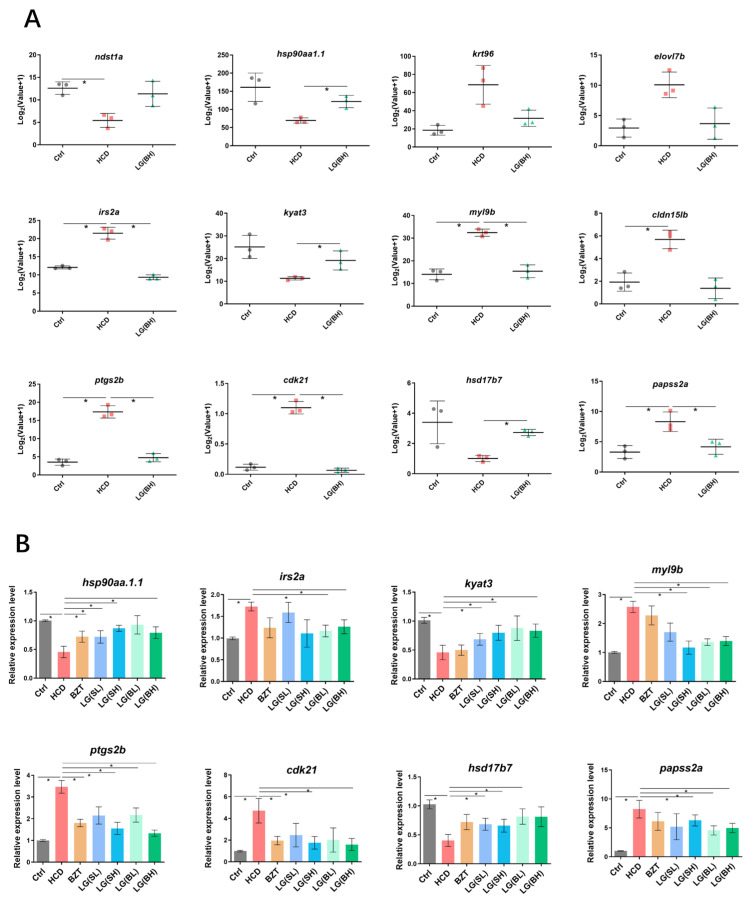
The expression of identified genes at key junction nodes in KEGG pathway relationship network. (**A**) The RNA-sequencing analysis of the expression of hsp90aa1.1, kyat3, hsd17b7, irs2a, myl9b, ptgs2b, cdk21 and papss2a in experimental groups. (**B**) Validation of screened genes by qRT-PCR test. Log2 (fold change) between treatment samples and control were calculated by the ^−∆∆CT^ method of three biologically independent replicates. Data are shown as the means ± SD. * *p* < 0.05.

Additionally, the expression of genes at key junction nodes was analyzed based on the RNA-sequencing results (Figure 6A). Of these genes, it was shown that hsp90aa1.1, kyat3, hsd17b7 were significantly upregulated in the *L. gasseri*-treated groups compared to the HCD model groups. Moreover, the expression of irs2a, myl9b, ptgs2b, cdk21 and papss2a were remarkably increased by HCD, and *L. gasseri* played critical roles in reducing their expression levels to normal stages. Afterward, these genes were selected to be subjected to an mRNA expression validation experiment with qRT-PCR (Figure 6B). The results confirmed that the expression levels of hsp90aa1.1, kyat3 and hsd17b7 were decreased after HCD treatment, and the lowering effect was reversed by LG administration. In contrast, the expression levels of irs2a, myl9b, ptgs2b, cdk21 and papss2a, which were increased after HCD induction, and were reduced to varying degrees by *L. gasseri*, indicating that these genes may potentially explain the inhibitory mechanism of *L. gasseri* on HCD-induced liver injury.

## 3. Discussion

Cholesterol accumulation is considered to be one of the factors contributing to the development of fatty liver [28]. Furthermore, recent studies have confirmed that a cholesterol-rich diet may cause liver inflammation and lowering cholesterol levels can ameliorate NASH, indicating that decreasing cholesterol levels might be used therapeutically [29,30]. Previous studies revealed a cholesterol-lowering potential of various lactobacilli, such as *L. acidophilus ATCC 4962*, *L. fermentum* [31,32], and *L. gasseri* SBT2055 [33]. In the high-fat model, *L. acidophilus NS1* was found to reduce obesity and liver lipid accumulation. At the same time, *L. acidophilus ATCC 43121* showed a positive effect on sterols excretion in feces [9,34]. Furthermore, the intake of *L. plantarum* and *L. fermentum MJM60397* decreased cholesterol levels in serum [35,36]. *L. gasseri* reduced total and low-density lipoprotein (LDL) cholesterol as well as triglycerides in rats [33]. *L. gasseri* increased fat droplet size, which is associated with lower absorption and digestion [37]. *L. gasseri* can also reinforce the excretion of acidic steroids in feces and decrease the reabsorption of bile acids [33]. The current research further confirmed that *L. gasseri* intake reduced cholesterol levels and reduced liver steatosis. Moreover, in this study, the ndst1 gene was downregulated in the HCD group, which is associated with the increased accumulation of triglyceride-rich lipoproteins [38]. All of these facts suggest that *L. gasseri* is a promising probiotic capable of ameliorating fatty liver progression.

One of the main contributors to the development of fatty liver is excessive intrahepatic triglyceride and lipid deposition [39]. The number of intrahepatic triglycerides was related to the triglyceride content, increased ratio of saturated and unsaturated fatty acids and lipolysis [40]. Lipolysis is an essential process for cell metabolism [41]. The dysregulation of this process contributes significantly to the generation of ROS and the progression of metabolic diseases, such as fatty liver [41]. According to previous research, patients with fatty liver had 50% higher rates of lipolysis [39]. The results obtained from our HCD zebrafish model also indicated increased rates of lipolysis, which corresponds to the previous reports. Additionally, we detected a decreased expression of the cholesterol biosynthesis-related gene hsd17b7 in the HCD group, which was partially relieved after *L. gasseri* treatment [42].

Probiotics are used to modulate the composition of the gut microbiome and contribute to various processes, such as the production of bacterial toxins, intestinal barrier fortification as well as the production of antimicrobial substances [43]. Probiotics also take part in the production of various anti-inflammatory cytokines and reduce pro-inflammatory cytokines, such as tumor necrosis factor-alpha (TNFα) [44,45,46]. Our results are in line with recent studies showing that treatment with *L. gasseri* has been shown to restore altered gut barrier function, as well as reduce inflammation in the intestine, lipid accumulation, and oxidative stress [47,48,49,50,51].

Oxidative stress plays a crucial role in fatty liver. The excessive production of reactive oxygen species (ROS) at the intestinal level and subsequent onset of oxidative stress contribute greatly to the damage of proteins, lipids, DNA and organelles, leading to inflammation, apoptosis and fatty liver progression [52,53]. For fatty liver, mitochondria dysfunction is the primary source of ROS, which is tightly connected to the endoplasmic reticulum stress [54]. Furthermore, lipid peroxidation and the production of MDA aggravate fatty liver by inducing liver steatosis [55]. The results obtained in current research showed that treatment with *L. gasseri* reduced ROS production and MDA content (Figure 3A, B) in HCD-fed zebrafish group. It corresponds to the findings of previous studies, where *L. gasseri* SBT2055 also showed protective effects against oxidative stress via activation of the Nrf2-ARE pathway in mammalian cells [56]. Additionally, *L. brevis* and *L. plantarum* were able to reduce intestinal oxidative stress and inflammation and contribute to intestinal barrier integrity in animal models [57,58]. Therefore, it is evident that probiotic consumption decreases the levels of oxidative stress via the regulation of antioxidant signaling pathways, stimulating the production of antioxidant metabolites (glutathione, butyrate, and folate) and inhibiting the ROS-producing enzymes [59,60]. Enhancing defenses against oxidative stress via probiotic consumption and the modulation of gut microbiota composition might be a promising strategy for the treatment of metabolic disorders.

IL-17 is a member of the crucial proinflammatory cytokine family [61]. IL-17 participates in the protection of the gut mucosa in HCD metabolic disorders and impacts the progression of fatty liver and NASH through the gut microbiota [61]. However, the exact nature of how it mediates the development of fatty liver remains unknown [61]. Our gene profiling results have confirmed high levels of IL-17 expression in the HCD-fed zebrafish group, indicating the connection between fatty liver development and IL-17 levels, which correlates with the findings of various research [62,63,64,65]. Gene ptgs2b (also known as COX-2), associated with inflammation and prostaglandins synthesis, was found to be related to the development of steatohepatitis in methionine- and choline-deficient (MCD) mice, was highly overexpressed in our HCD fed zebrafish group [66]. Recent studies revealed that IL-17 limits the excessive permeability of the gut barrier through the IL-17 receptor adaptor protein Act-1 [67]. The study of He et al. has further confirmed that IL-17 deficient mice showed disturbed intestinal barrier and altered GM, which promoted the development of fatty liver [61]. On the contrary, the latest Mendelian randomization study showed that IL-17 was related to the increased risk of fatty liver development [68]. Additionally, IL-17 showed an effect on the GM composition and fatty liver in an HFD mouse model via the yet unknown mechanism [69]. In this work, the levels of ptgs2b decreased drastically after treatment with *L. gasseri*. Together, these studies suggest a close relationship between IL-17 and fatty liver. Further research is required to determine the role of IL-17 in fatty liver development and progression.

Tumor-suppressor factor p53 has been studied for years and has become a point of interest for liver disease research due to its role in multiple signaling pathways that induce apoptosis, cell cycle arrest, cell differentiation and angiogenesis [70,71]. The latest research revealed that p53 has a major function in the development of fatty liver [72,73,74]. P53 contributes to the accumulation of lipid droplets [75] and lipid metabolism [76,77,78] and promotes the development of obesity by regulating adipose tissue differentiation [79]. Furthermore, there is a growing amount of evidence that p53 is involved in various forms of hepatocellular injury [80,81,82,83,84]. Our findings further confirmed elevated p53 signaling pathways in the HCD zebrafish model (Figure 4D). Thus, it is clear that p53 is associated with fatty liver; however, several previous studies obtained controversial results. A number of studies have, in fact, shown that activated p53 is essential to fatty liver development [76,83,85], while others pointed out that inhibition of p53 led to the aggravation of liver steatosis [81,86,87,88,89].

Estrogens contribute to the regulation of multiple organ systems, as well as the regulation of liver lipid metabolism [90]. According to the results obtained from a transgenic mouse model, the liver showed the highest response to estrogen [91]. Thus, estrogen deficiency can lead to increased liver lipid deposition and disturbances in liver energy metabolism [92]. Mostly, estrogen performs its functions through the interaction with steroid nuclear hormone receptors, estrogen receptor alpha (Erα) or estrogen receptor beta (ERβ) [90]. Unliganded ERα and ERβ bind to Hsp90. Hsp90, together with p23 and immunophilin, form a large molecular complex to keep ERα stable and inactive [93]. The inhibition of Hsp90 leads to ERα ubiquitination and subsequent proteasomal degradation [94,95]. Our findings revealed the downregulation of Hsp90aa.1 gene in the HCD zebrafish group, which indicates that Estrogen action is disrupted in fatty liver disease. After treatment with *L. gasseri*, the expression level of Hsp90aa.1 was increased.

Overall, this study demonstrated that *L. gasseri* attenuated fatty liver progression by the attenuation of oxidative stress, as well as lipid accumulation and inflammation, and regulation of host gene expression profiles. Therefore, *L. gasseri* is a promising probiotic that can be used for the treatment of fatty liver.

## 4. Materials and Methods

### 4.1. Bacteria Cultivation

Lactobacillus gasseri M01 GDMCC60781 (*L. gasseri M01*) was a laboratory-conserved strain deposited in GDMCC (Guangdong Microbial Culture Collection Center, Guangzhou, China) [96]. In this research, *L. gasseri M01* was revived, activated and grown in Man Rogosa Sharpe (MRS) broth at 37 °C for 12 h in accordance with the previous study [96].

### 4.2. Zebrafish Husbandry

Zebrafish were raised in compliance with the compliance with Institutional Animal Care and Use Committee (IACUC) guidelines of Nanjing Tech University for laboratory animal use. Adult wild-type zebrafish (Tübingen line) were obtained from Nanjing Yaoshunyu Biotechnology Co., Ltd. (Nanjing, China) and maintained in accordance with the standard procedures as described previously [97]. Transgenic zebrafish embryos with macrophages labeled with mCherry, *Tg (mpx:mCherry)* were provided by China Zebrafish Research Center (CZRC). Transgenic zebrafish embryos *Tg (lfabp:dsRed)* were gifted from Professor Mingfang He’s lab (School of Biological and Pharmaceutical Engineering, Nanjing Tech University, Nanjing, China). Fish spawning was implemented artificially by light stimulation, with the male–female mating ratio of 1:2. All of the embryos were maintained in a closed flow-through system with 14 h–10 h light-dark cycle at a temperature of 28 ± 0.5 °C [98]. The embryos were not used for experimental treatments when the fertilization rate was lower than 85%.

### 4.3. Preparation of High Cholesterol Diet and Bacteria

Cholesterol (purity: ≥92.5%) was purchased from Sigma-Aldrich (St. Louis, MO, USA). Bezafibrate (purity: ≥98%, BZT) was obtained from Aladdin (Shanghai, China). BZT was the positive control drug, which was dissolved in dimethyl sulfoxide (DMSO, Nanjing Chemical Reagent Co. Ltd., Nanjing, China) with water to a final concentration of 10 µM/L (DMSO 0.001% *v*/*v*) [55]. Zebrafish larvae, after three days fertilization, were fed basic food, paramecium culture and AP100 (Shanghai FishBio Co., Ltd., Shanghai, China) daily. The HCD was prepared by mixing cholesterol and basic food in ether (2 mL ether per gram of food) and then using a 60 °C water bath to volatilize the ether of the HCD [55]. The final cholesterol concentration in HCD was 4% (*w*/*w*) [99]. The present study was carried out with both the cells (*L. gasseri* suspension) and the cell-free culture supernatants (*L. gasseri* breaking supernatant). The *L. gasseri* suspension was prepared by centrifuging the *L. gasseri* culturing medium at 4000 rpm for 5 min, discarding the supernatant, washing the cells 3 times with phosphate-buffered saline (PBS) and then 3 times with zebrafish culture water; *L. gasseri* breaking supernatant was prepared by ultrasonic treatment of *L. gasseri* suspension. The ultrasonic treatment time was 25 min, with 5 s pauses every 3 s. Then, the supernatant was collected by centrifuge at 12,000× *g* rpm for 10 min. The *L. gasseri* suspension and *L. gasseri* breaking supernatant were diluted to 10^6^ and 10^7^ CFU/mL with culture water as low and high concentrations, respectively.

### 4.4. Grouping of Experimental Animals

Eight-day post-fertilization (dpf) zebrafish larvae were randomly divided into 7 groups (*n* = 100 per group) and fed different diets as follows: (1) Control (Ctrl), AP100 (20 mg/tank per day); (2) high cholesterol diet treated groups (HCD), AP100 (20 mg/tank per day) and 4% (*w*/*w*) cholesterol; (3) Bezafibrate-treated groups (BZT), AP100 (20 mg/tank per day), 4% (*w*/*w*) cholesterol and 10 µM/L BZT; (4) *L. gasseri* suspension groups with low concentration of bacteria (LG (SL)), AP100 (20 mg/tank per day), 4% (*w*/*w*) cholesterol and 10^6^ CFU/mL suspension of *L. gasseri*; (5) LG (SH) groups, AP100 (20 mg/tank per day), 4% (*w*/*w*) cholesterol and 10^7^ CFU/mL suspension of *L. gasseri*; (6) LG (BL) groups, AP100 (20 mg/tank per day), 4% (*w*/*w*) cholesterol and 10^6^ CFU/mL breaking supernatant of *L. gasseri;* (7) LG (BH) groups, AP100 (20 mg/tank per day), 4% (*w*/*w*) cholesterol and 10^7^ CFU/mL breaking supernatant of *L. gasseri*. All of the groups were maintained following the schedule of Figure 1A.

### 4.5. Biochemical Analysis

The content of the malondialdehyde (MDA) levels was measured using a commercial assay kit (Nanjing Jiancheng Bioengineering Institute, Nanjing, China), following the manufacturer’s instructions. The quantitation of reactive oxygen species (ROS) was detected with the Reactive Oxygen Species Assay Kit (Beyotime Biotechnology, Shanghai, China), according to the manufacturer’s instructions. Briefly, 30 zebrafish larvae from the groups above were defrosted and homogenized with 10 volumes of iced PBS (pH 7.4) for 20 min. The homogenate was then centrifuged by cryogenic centrifuge (Thermo Fisher Scientific, Waltham, MA, USA) at 4000× *g* rpm for 5 min, and the supernatant was collected for the biochemical tests. All of the measurements were repeated three times.

### 4.6. Histopathological Analysis

Zebrafish larvae were fixed with 4% paraformaldehyde in phosphate-buffered saline (P6748, Sigma-Aldrich, St. Louis, MO, USA) and paraffin-embedded. Microtome was used to slice the larvae into 5 μm thickness sections, and the slices were stained with hematoxylin and eosin (H&E). H&E (H-3404, Vector labs) staining was carried out following the manufacturer’s protocols. The pathological features of histopathology were analyzed based on the previous study [100]. At least five sections from each treatment group were microphotographed using an inverted microscope (Nikon ECLIPSE Ts2, Tokyo, Japan).

### 4.7. Nile Red Staining

Nile red is a lipophilic fluorescent substance that labels triglycerides and fatty acids with red fluorescence. In this study, Nile red (purity: ≥95%) purchased from Macklin (Shanghai, China) was dissolved with acetone to 2 mg/mL and then diluted with water to the final concentration of 1 mg/mL. The zebrafish larvae were stained with the Nile red solution in the dark for 30 min and washed with water three times. After cleaning, the zebrafish larvae were anesthetized with 0.05% tricaine and kept in sodium carboxymethyl cellulose (CMC-Na) (4%). The photomicrograph was immediately performed under an emission maximum of about 638 nm using a fluorescence stereoscope (Nikon ECLIPSE Ts2, Tokyo, Japan).

### 4.8. Quantification of Reactive Oxygen Species (ROS)

For in vivo ROS detection, 15 living zebrafish larvae at 7 dpf in each group were randomly collected and stained with 100 μM of 2′,7′-dichlorodihydrofluorescein (DCFH-DA, Beyotime Biotechnology, Shanghai, China) for 40 min, following the instructions. After rinsing with water three times for 5 min each, at diacetate, at least 10 fish from each group were photographed at the excitation of 488 nm wavelength (Nikon ECLIPSE Ti2E, Tokyo, Japan).

### 4.9. Real-Time Quantitative PCR (qRT-PCR) Analysis

A total of 30 zebrafish larvae of each group were sacrificed to extract the total RNA using Trizol reagent (Invitrogen, Carlsbad, CA, USA) and purified using the RNease Mini Kit (Qiagen, Valencia, CA, USA). The concentrations of the total RNA were quantified using a nucleic acid and protein spectrophotometer (Nano-300, Aosheng Instrument Co., Ltd., Hangzhou, China). HiScript II qRT SuperMix (Vazyme, Nanjing, China) was used to perform the reverse transcription for cDNA synthesis. The qPCR was implemented on the StepOnePlus Real-Time PCR System (Applied Biosystems, Foster City, CA, USA) by adding the ChamQTM Universal SYBR qPCR Master Mix (Vazyme, Nanjing, China) following the manufacturer’s protocol. The qPCR was performed with 2 min for template annealing at 95 °C, 40 cycles of amplification at 95 °C for 10 s, 60 °C for 30 s, and a final extension at 95 °C for 15 s and 60 °C for 2 min. Primers (Table 1) were designed using PRIMER 5 software and purchased from GenScript, Nanjing China. The 2^−∆∆Ct^ method was used to calculate the expression levels of each targeting mRNA by normalizing it to glyceraldehyde-3-phosphate dehydrogenase (Gapdh) [101]. All of the samples were determined in triplicate.

### 4.10. RNA Transcriptomic Analysis

The total RNA was isolated, and the RNA quality was assessed using an Agilent 2100 Bioanalyzer (Agilent Technologies, Palo Alto, CA, USA). RNA sequencing was implemented after the construction of the library using the TruSeq PE Cluster Kit V3-cBot-HS (Illumina, NEB, USA). All libraries were loaded into the same lane of the HiSeq 2000 (TruSeq SBS KIT-HS V3, Illumina, Santa Clara, CA, USA) at Beijing Genomics Institute (Shenzhen, China). The raw reads obtained were filtered through SOAPnuke (v1.5.2) to remove low-quality reads and linker sequences. The clean reads were mapped to the zebrafish reference sequence database (Danio rerio, UCSC version danRer7, 2010) with TopHat2.0 (http://ccb.jhu.edu/software/tophat/index.shtml, accessed on 2 December 2020) [102]. DESeq2 (v1.4.5) was used to identify the differentially expressed genes (DEGs) between groups. The parameters for assessing transcripts with differential expression were Q value <  0.05 and |log2FC|  >  1 [103]. The heatmap was generated with Heatmap (v1.0.8) according to the identified differentially expressed genes in each sample. The DEGs were analyzed with GO (http://www.geneontology.org/, accessed on 7 December 2020), the Database for Annotation, Visualization and Integrated Discovery (DAVID), KEGG (http://www.kegg.jp/kegg/pathway.html, accessed on 7 December 2020) enrichment annotation and BGI online system, Dr. Tom (BGI-Shenzhen, Shenzhen, China) to take insight to the changes in gene expression and signaling.

### 4.11. Statistical Analysis

All of the experiments were repeated independently at least three times. Statistical analysis was performed through GraphPad Prism 6 (GraphPad Software, San Diego, CA, USA). The difference between the treatment groups and the corresponding control was compared by one-way analysis of variance (ANOVA) with Tukey’s honestly significant difference test (Tukey’s HSD) at the significance *p*-value < 0.05. All of the data were presented as mean values ± standard deviation (SD). The fluorescence intensity was evaluated with digital images produced using the Image J software (developed by the National Institutes of Health) as described [104].

## Figures and Tables

**Table 1:** Primers sequences.

Gene	Gene ID	Forward Sequence	Reverse Sequence
*papss2a*	777719	GCAAGTTGTAGGCACCAGAG	CCATGACGGATGTTGTCACC
*hsd17b7*	768185	TAGCTTAGCCTTGAACCGCC	TGGTATTTGGCCATCGGGTC
*cdk21*	569515	CTGAAGCCTGACAATGTGCT	GCAAGCCAATTACCTCAAAGA
*myl9b*	406493	CTGCTTCGATGAGGAGGGAT	GGCTCCGTGTTTGAGGATTC
*iras2a*	393285	CTCCGAGGTGGCATCAGTTAC	CCCCTTCACTTGCAGTCCGTATT
*ptgs2b*	559020	CCCCAGAGTACTGGAAACCA	ACATGGCCCGTTGACATTAT
*hsp90aa1.1*	30591	AGGCCTTTGTGCCGGATTTA	TTGCAGTGGGGTGTTTATGC
*kyat3*	393315	TGGAATGTGGGACAGAACCA	GCCAACAGCCTCCTGTAATG
*gapdh*	317743	CCAACTGCCTGGCTCCTT	CCCATCAACGGTCTTCTGTG

## Data Availability

The data that to support the findings of this study are available from the corresponding author upon request.

## Supplementary Materials

The following supporting information can be downloaded at: https://www.mdpi.com/article/10.3390/ijms24032053/s1.
