# Supplementary material for: Protective Effects of Lactobacillus gasseri against High-Cholesterol Diet-Induced Fatty Liver and Regulation of Host Gene Expression Profiles
Source: Int J Mol Sci. 2023 Jan 20;24(3):2053. doi: 10.3390/ijms24032053 (PMC9917166; doi:10.3390/ijms24032053)
Supplement: Supplementary file 1 [file ijms-24-02053-s001.zip › ijms-2148013-supplementary.pdf]

Table S1. DEGs upregulated by HCD and downregulated after the treatment with *L. gasseri*.

Gene ID	Gene Symbol	log2 (LG(BH) / HCD)	Q value (LG(BH) / HCD)	P value LG(BH)/ HCD	log2 (HCD / Ctrl)	Q value (HCD /Ctrl)	P value (HCD/ Ctrl)
**100000683**	'si:ch211-131e11.19'	-2.93757	1.32E-05	2.12E-08	3.127415	1.63E-05	3.57E-08
**100002043**	'LOC100002043'	-7.63016	1.67E-06	1.75E-09	5.108741	5.17E-05	1.41E-07
**100002541**	'zgc:172053'	-1.64595	0.030054	2.92E-04	2.81076	9.71E-05	3.00E-07
**100003687**	'LOC100003687'	-3.02264	9.48E-07	7.39E-10	1.85826	0.004549	3.44E-05
**100004603**	'si:ch73-14h1.2'	-2.57751	1.54E-11	3.00E-15	1.462356	0.013777	1.39E-04
**100004951**	'LOC100004951'	-5.27646	6.35E-04	1.95E-06	3.43254	0.024456	2.87E-04
**100006263**	'si:dkey-23a13.11'	-4.70019	0.01448	1.08E-04	4.617454	0.016022	1.67E-04
**100006469**	'LOC100006469'	-0.99676	0.005688	3.08E-05	1.17708	0.009557	8.81E-05
**100006523**	'zgc:198419'	-0.91139	0.015125	1.15E-04	1.062408	0.005121	3.98E-05
**100009638**	'zgc:153932'	-1.25255	9.95E-06	1.41E-08	1.276356	0.001918	1.21E-05
**100126807**	'zgc:171242'	-2.78857	5.71E-05	1.16E-07	3.02546	1.08E-04	3.45E-07
**100144555**	'btr22'	-2.91209	4.48E-17	1.75E-21	1.797355	1.53E-06	2.51E-09
**100148920**	'LOC100148920'	-7.62425	1.19E-08	5.57E-12	7.540416	1.54E-08	1.80E-11
**100331068**	'LOC100331068'	-5.48533	0.024456	2.27E-04	5.394369	0.032333	4.18E-04
**100331080**	'LOC100331080'	-1.1574	1.45E-06	1.36E-09	0.938304	0.030683	3.87E-04
**100331359**	'LOC100331359'	-6.78739	6.11E-04	1.86E-06	5.734636	0.007745	6.83E-05
**100331388**	'si:ch73-158p21.7'	-1.80772	1.20E-06	1.03E-09	1.175793	0.01677	1.80E-04
**100331822**	'micall1b.2'	-2.20294	0.008566	5.44E-05	2.814139	3.68E-04	1.58E-06
**100332428**	'LOC100332428'	-1.38352	0.026777	2.55E-04	1.453327	0.049479	7.67E-04
**100332647**	'si:ch211-198m17.1'	-1.16121	8.29E-04	2.71E-06	0.940695	0.044014	6.56E-04
**100333484**	'LOC100333484'	-2.34984	1.65E-06	1.64E-09	3.032886	2.45E-07	3.54E-10
**100534759**	'si:dkey-22n8.3'	-1.36654	6.64E-04	2.07E-06	2.440613	7.49E-10	5.85E-13
**100535405**	'LOC100535405'	-2.65176	1.18E-05	1.83E-08	2.65271	4.18E-04	1.94E-06
**100536895**	'si:ch211-181d7.3'	-1.45933	3.44E-09	1.48E-12	2.716078	9.29E-21	7.53E-25
**101882272**	'LOC101882272'	-1.13458	0.002487	1.09E-05	1.553169	8.51E-04	4.42E-06
**101882496**	'LOC101882496'	-9.06178	3.44E-09	1.46E-12	8.970863	4.54E-09	4.43E-12
**101883708**	'LOC101883708'	-2.18163	2.65E-11	6.19E-15	2.4858	3.65E-08	4.41E-11
**101883788**	'LOC101883788'	-1.99338	2.20E-06	2.57E-09	3.030382	9.34E-12	5.47E-15
**101883994**	'LOC101883994'	-1.89985	0.006524	3.79E-05	2.145539	0.003377	2.29E-05
**101885252**	'LOC101885252'	-1.30098	7.92E-04	2.53E-06	1.845066	6.70E-08	9.15E-11
**101885678**	'LOC101885678'	-3.80873	2.81E-05	5.36E-08	2.439103	0.002422	1.56E-05
**101885950**	'LOC101885950'	-4.38586	2.12E-12	2.48E-16	2.177453	3.73E-04	1.64E-06
**101886086**	'LOC101886086'	-3.56587	2.71E-04	7.08E-07	3.32814	0.001877	1.17E-05
**103908677**	'LOC103908677'	-1.69094	0.041882	4.52E-04	2.45709	0.001827	1.12E-05
**103909212**	'LOC103909212'	-0.81135	0.019956	1.67E-04	1.378182	7.08E-07	1.08E-09

**103909796**	'LOC103909796'	-1.42891	2.68E-08	1.36E-11	1.591502	3.15E-04	1.25E-06
**103910140**	'LOC103910140'	-1.12436	0.009166	5.93E-05	2.525168	3.98E-09	3.73E-12
**103910211**	'LOC103910211'	-5.41449	0.0356	3.66E-04	5.323396	0.044267	6.62E-04
**103911469**	'LOC103911469'	-5.58392	0.030782	3.02E-04	5.493373	0.039671	5.51E-04
**108004537**	'si:ch73-196i15.3'	-2.92948	1.48E-05	2.42E-08	2.111609	0.003841	2.67E-05
**108179099**	'LOC108179099'	-5.70017	0.004695	2.39E-05	5.609337	0.005432	4.35E-05
**108179116**	'LOC108179116'	-1.90639	8.73E-12	1.36E-15	1.121837	0.011141	1.06E-04
**108180185**	'LOC108180185'	-5.83313	0.001166	4.23E-06	5.181145	0.00135	7.90E-06
**108182767**	'LOC108182767'	-2.11385	0.008591	5.49E-05	2.267572	0.003971	2.79E-05
**108182798**	'LOC108182798'	-2.23398	0.006603	3.89E-05	2.353125	0.03436	4.55E-04
**108190121**	'LOC108190121'	-1.49416	1.52E-04	3.66E-07	-1.26311	0.00135	7.82E-06
**110437703**	'LOC110437703'	-1.40925	2.13E-04	5.40E-07	2.031322	1.09E-05	2.34E-08
**110437747**	'LOC110437747'	-1.27118	9.28E-06	1.27E-08	2.38696	8.77E-14	3.42E-17
**110437770**	'LOC110437770'	-0.95342	3.29E-04	9.23E-07	1.202893	0.001877	1.17E-05
**110437982**	'LOC110437982'	-1.4542	0.001106	3.92E-06	-1.25705	0.004241	3.11E-05
**110438671**	'LOC110438671'	-1.36227	0.048942	5.59E-04	2.461798	6.34E-04	3.09E-06
**110438850**	'LOC110438850'	-7.64318	3.59E-06	4.47E-09	6.590484	1.56E-04	5.60E-07
**110438910**	'LOC110438910'	-5.57164	0.026369	2.49E-04	6.442902	0.005897	4.87E-05
**110439066**	'LOC110439066'	-3.36703	0.019956	1.67E-04	3.021627	0.039013	5.36E-04
**110439162**	'LOC110439162'	-2.08813	0.002956	1.37E-05	2.740892	1.46E-04	5.02E-07
**110439296**	'LOC110439296'	-2.65864	0.007738	4.86E-05	3.665048	0.001097	6.17E-06
**110439553**	'LOC110439553'	-1.92603	0.021691	1.92E-04	3.362162	3.76E-04	1.69E-06
**110440005**	'LOC110440005'	-3.33301	0.002517	1.11E-05	3.081851	0.004161	3.01E-05
**110440062**	'LOC110440062'	-5.64145	0.015867	1.22E-04	5.550227	0.020721	2.30E-04
**321502**	'krt96'	-1.09472	0.00209	8.96E-06	1.862608	5.33E-08	6.87E-11
**327253**	'hdac10'	-1.11007	0.006336	3.59E-05	1.204857	0.012422	1.23E-04
**327274**	'elovl7b'	-1.463	0.036014	3.73E-04	1.741507	0.00135	7.87E-06
**337770**	'si:ch73-187m15.4'	-7.44402	4.27E-13	3.33E-17	7.629067	5.96E-14	2.10E-17
**393285**	'irs2a'	-1.52794	1.03E-06	8.43E-10	1.132035	0.00471	3.60E-05
**393297**	'zgc:56585'	-1.44446	0.030753	3.00E-04	1.869165	0.001877	1.17E-05
**393483**	'tmem150ab'	-2.78561	1.85E-09	5.76E-13	2.877264	1.47E-08	1.61E-11
**403338**	'gnmt'	-0.97285	0.001239	4.63E-06	1.352803	0.015675	1.62E-04
**405810**	'ehd1a'	-0.76275	0.020395	1.76E-04	0.902782	0.039134	5.40E-04
**405845**	'zgc:77118'	-4.7338	0.001239	4.69E-06	5.251512	0.002803	1.85E-05
**406493**	'myl9b'	-1.06388	8.90E-06	1.18E-08	1.298324	4.18E-04	1.94E-06
**406630**	'cmc1'	-1.31084	1.89E-05	3.40E-08	1.272334	3.73E-04	1.62E-06
**407650**	'rabac1'	-0.81018	0.008359	5.28E-05	0.917263	0.006217	5.19E-05
**436616**	'zgc:92184'	-10.5573	6.85E-11	1.87E-14	9.503924	3.98E-11	2.95E-14
**436719**	'cldn15lb'	-1.93453	0.005713	3.12E-05	1.723721	0.038035	5.14E-04
**541383**	'ms4a17a.9'	-1.08551	0.019956	1.67E-04	1.334044	0.010207	9.60E-05
**541543**	'zgc:112970'	-5.42908	1.11E-05	1.65E-08	3.901954	6.51E-05	1.86E-07
**553622**	'phlda2'	-0.89831	0.025201	2.35E-04	1.301273	8.26E-04	4.25E-06
**553761**	'arhgef3l'	-1.5328	0.039135	4.12E-04	1.618461	0.042854	6.19E-04

**556912**	'LOC556912'	-6.61476	3.08E-04	8.52E-07	6.524029	4.06E-04	1.85E-06
**557131**	'si:ch211-285c6.3'	-6.00116	0.006585	3.85E-05	5.910422	0.008649	7.70E-05
**557570**	'si:dkeyp-41f9.3'	-1.22683	0.013812	1.00E-04	1.436497	0.004153	2.95E-05
**558816**	'LOC558816'	-1.3309	5.49E-04	1.61E-06	0.988369	0.045439	6.88E-04
**559020**	'ptgs2b'	-1.4824	6.00E-04	1.80E-06	2.237942	6.82E-13	2.93E-16
**559160**	'si:ch211-214j24.14'	-1.06428	9.49E-04	3.18E-06	1.032405	0.023899	2.79E-04
**563091**	'tnni4b.1'	-1.40479	0.009782	6.50E-05	1.354368	0.009641	8.98E-05
**564660**	'LOC564660'	-1.79102	0.03178	3.17E-04	1.943452	0.020863	2.34E-04
**565387**	'tmem104'	-0.88842	0.018759	1.53E-04	0.917377	0.039691	5.55E-04
**566574**	'LOC566574'	-5.74348	0.031929	3.20E-04	5.652837	0.042124	5.99E-04
**567247**	'mier3b'	-1.18162	5.26E-05	1.05E-07	1.306528	3.94E-05	1.01E-07
**567883**	'LOC567883'	-1.21389	4.40E-07	2.92E-10	1.328958	4.33E-06	8.28E-09
**569515**	'cdk21'	-3.73355	9.35E-04	3.10E-06	2.9129	0.006895	5.95E-05
**570070**	'kcnh6b'	-1.50129	0.01859	1.51E-04	2.035367	0.001174	6.73E-06
**570991**	'si:ch1073-340i21.3'	-3.71287	3.08E-06	3.72E-09	5.075324	4.27E-06	7.92E-09
**572245**	'noxo1a'	-1.91381	0.009782	6.52E-05	2.233592	3.22E-04	1.32E-06
**641575**	'actn2b'	-1.169	1.18E-05	1.83E-08	1.193309	1.43E-04	4.79E-07
**723999**	'zgc:136333'	-1.47952	0.001087	3.81E-06	1.691018	9.57E-04	5.15E-06
**751765**	'pmaip1'	-1.65978	1.79E-06	2.03E-09	2.304298	2.18E-09	1.87E-12
**777719**	'papss2a'	-0.96925	0.021179	1.87E-04	1.304881	0.001426	8.46E-06
**792625**	'cplane1'	-0.99195	0.001018	3.53E-06	0.942645	0.009557	8.84E-05
**794920**	'nectin4b'	-0.88246	0.049686	5.71E-04	0.939388	0.049479	7.71E-04
**795785**	'cxcl18b'	-1.21207	0.001166	4.19E-06	2.847983	3.30E-12	1.74E-15
**797491**	'itgb7'	-3.30633	2.82E-04	7.58E-07	3.22415	4.76E-04	2.25E-06
**797677**	'si:dkey-247k7.2'	-1.07347	6.33E-05	1.31E-07	1.079944	0.031282	3.99E-04
**797963**	'LOC797963'	-6.14456	0.004695	2.38E-05	6.053878	0.005886	4.81E-05
**798290**	'ms4a17a.2'	-1.99628	7.17E-04	2.26E-06	3.021416	4.27E-06	8.00E-09
**799627**	'ptpn21'	-1.23057	0.048349	5.46E-04	1.686481	0.004762	3.66E-05

Table S2. DEGs downregulated in HCD but upregulated after *L. gasseri* treatment.

Gene ID	Gene Symbol	log2 (LG （BH） / H)	Qvalue (LG （BH） / HCD)	Pvalue(LG （BH） / H)	log2 (HCD / Ctrl)	Qvalue (HCD /Ctrl)	Pvalue(LG （BH） / T)
**100002785**	'ms4a17a.8'	1.97048	1.52E-04	3.62E-07	-1.90313	3.15E-04	1.27E-06
**100034563**	'si:rp71-36a1.3'	2.581207	0.013465	9.66E-05	-3.9066	6.28E-08	8.34E-11
**100141355**	'zgc:171497'	1.525202	0.001838	7.53E-06	-3.02862	9.29E-21	1.09E-24
**100142642**	'sc:d217'	2.016945	2.13E-04	5.39E-07	-2.87881	1.62E-11	1.01E-14
**100319138**	'ipo13'	1.12093	2.88E-05	5.61E-08	-0.84768	0.025328	3.00E-04
**100329944**	'ndst1a'	1.06447	0.02313	2.11E-04	-1.14348	0.022762	2.63E-04
**100526651**	'tcf4'	1.303973	0.041449	4.41E-04	-1.94651	0.006361	5.39E-05
**100535018**	'uimc1'	0.826699	0.001371	5.34E-06	-0.66998	0.047128	7.19E-04
**100535070**	'LOC100535070'	1.722767	8.55E-08	5.00E-11	-2.19463	3.13E-07	4.64E-10
**100535478**	'si:ch211-194e18.2'	0.764877	0.035547	3.64E-04	-1.07473	0.007244	6.36E-05
**101882166**	'LOC101882166'	1.225866	0.001455	5.90E-06	-1.0962	0.046954	7.15E-04

**101884921**	'LOC101884921'	0.676411	0.011333	7.82E-05	-0.99961	2.20E-05	5.25E-08
**101885874**	'LOC101885874'	1.22859	0.01156	8.11E-05	-1.57624	8.12E-04	4.14E-06
**101886784**	'LOC101886784'	2.227815	1.65E-06	1.67E-09	-2.37363	7.98E-09	8.10E-12
**101887118**	'LOC101887118'	1.477442	0.002874	1.32E-05	-1.78337	2.95E-04	1.15E-06
**103909472**	'LOC103909472'	1.36514	0.00209	8.91E-06	-1.5921	0.001784	1.09E-05
**108183933**	'LOC108183933'	2.018772	3.00E-04	8.18E-07	-1.87032	0.002371	1.52E-05
**108190952**	'LOC108190952'	2.088157	1.76E-04	4.33E-07	-1.83476	0.022212	2.52E-04
**110437790**	'LOC110437790'	1.24403	0.042709	4.64E-04	-1.96807	1.09E-04	3.53E-07
**110438294**	'LOC110438294'	0.916194	0.005224	2.69E-05	-1.00452	0.009557	8.77E-05
**110438911**	'LOC110438911'	2.590241	0.019772	1.63E-04	-3.01072	2.08E-04	7.94E-07
**30591**	'hsp90aa1.1'	0.824851	0.002517	1.12E-05	-1.22529	1.31E-04	4.31E-07
**393315**	'kyat3'	0.753099	0.026859	2.58E-04	-1.14082	6.58E-04	3.26E-06
**436682**	'rbpms2a'	0.59953	0.027978	2.70E-04	-0.90222	0.009557	8.84E-05
**447816**	'rtn4a'	0.672305	0.043368	4.77E-04	-0.77228	0.016022	1.67E-04
**494041**	'slc7a2'	1.392446	0.004377	2.15E-05	-1.49612	0.003549	2.44E-05
**553267**	'elmod2'	0.605936	0.031976	3.22E-04	-0.92929	0.006303	5.31E-05
**558785**	'si:dkey-73p2.1'	1.55726	0.00698	4.16E-05	-1.74326	0.005886	4.82E-05
**566121**	'si:ch211-114l13.1'	1.039379	9.98E-04	3.39E-06	-1.19718	0.002886	1.93E-05
**568616**	'smyd2b'	1.474894	5.28E-07	3.70E-10	-1.31862	0.025493	3.02E-04
**641497**	'eefsec'	1.07098	0.004177	2.02E-05	-1.12069	0.008556	7.58E-05
**678556**	'cldn15b'	1.161831	0.015867	1.21E-04	-1.29798	0.007196	6.29E-05
**768185**	'hsd17b7'	1.441309	0.026501	2.51E-04	-1.75427	0.016652	1.77E-04
**795735**	'LOC795735'	2.019525	1.52E-04	3.64E-07	-1.87573	0.020863	2.34E-04
